# Antiviral Agents From Fungi: Diversity, Mechanisms and Potential Applications

**DOI:** 10.3389/fmicb.2018.02325

**Published:** 2018-10-02

**Authors:** Riikka Linnakoski, Dhanik Reshamwala, Pyry Veteli, Marta Cortina-Escribano, Henri Vanhanen, Varpu Marjomäki

**Affiliations:** ^1^Natural Resources Institute Finland (Luke), Helsinki, Finland; ^2^Division of Cell and Molecular Biology, Department of Biological and Environmental Science, Nanoscience Center, University of Jyväskylä, Jyväskylä, Finland; ^3^Natural Resources Institute Finland (Luke), Joensuu, Finland

**Keywords:** antiviral agents, antiviral mechanisms, endophytes, fungal secondary metabolites, medicinal mushrooms, natural products

## Abstract

Viral infections are amongst the most common diseases affecting people worldwide. New viruses emerge all the time and presently we have limited number of vaccines and only few antivirals to combat viral diseases. Fungi represent a vast source of bioactive molecules, which could potentially be used as antivirals in the future. Here, we have summarized the current knowledge of fungi as producers of antiviral compounds and discuss their potential applications. In particular, we have investigated how the antiviral action has been assessed and what is known about the molecular mechanisms and actual targets. Furthermore, we highlight the importance of accurate fungal species identification on antiviral and other natural products studies.

## Introduction

Viruses cause serious outbreaks in all continents leading to difficult symptoms and mortality, and enormous economic burden for society. In addition, the constant emergence of new serotypes in virus groups that have a high mutation rate and low fidelity for viral replication adds challenges in combatting against these viruses.

Viruses can be divided into those containing a lipid envelope and those whose genome is only covered by a protein shell. Enveloped viruses are less stable and more prone to degradation when treated with lipid solvents. Their infection mechanisms are usually based on the presence of fusogenic peptides in the lipid envelope leading to a merge of viral and cellular membranes. The non-enveloped viruses are much more stable and may stay active in wastewaters and on surfaces from several weeks to months. The non-enveloped viruses such as Noro viruses and enteroviruses are therefore causing outbreaks that are difficult to handle. In addition, they show little sensitivity to chemical disinfectants (Wutzler and Sauerbrei, 2004; Chan and Abu Bakar, 2005). Thus, there is a need for both vaccines and antivirals to encounter viral infections. However, the development of vaccines against a wide range of newly emerging virus serotypes is challenging, and currently vaccines are available only against a handful of viruses. In addition, vaccination cannot help if the infection is already present in the system.

The antiviral drugs inhibit the virus infection either by specifically targeting the viral proteins or the host cellular factors that the viruses exploit for their reproduction (Clercq, 2002). However, the problem in using viral proteins as drug targets is the high rate at which viruses produce mutant resistant strains against them (De Palma et al., 2008). Cellular factors exploited by viruses also serve as potential drug targets. However, they cannot be considered automatically as reliable targets, since viruses may deviate from their original pathway and still cause an effective infection (Van der Linden et al., 2015). Also, targeting cellular factors might have an adverse effect on normal functioning of the host cells. Furthermore, the mechanisms of non-enveloped viruses to break the host cell membrane barrier is less well known, which forms an additional challenge in developing strategies against these viruses.

An antiviral drug has to fulfill a set of prerequisites when undergoing preclinical and clinical trials. A vital requirement is that the drug should be effective in inhibiting the virus infection without causing any cytotoxicity and with minimal side effects to the host cells. In addition, a drug should be able to completely inhibit the virus infection, partial inhibition leads to the generation of drug resistant mutant strains. Due to these prerequisites, only a handful of synthetic antiviral drugs have made it past the clinical phase. Until today, the successful ‘one bug–one drug’ approach has been used for antiviral drug development. However, today the focus has shifted toward designing broad-spectrum antivirals, which can act on multiple viruses by targeting a common but essential viral function (Vigant et al., 2015). Combinatorial chemistry is nowadays a preferred approach adapted by major drug companies for discovering pharmacologically significant compounds (Strobel and Daisy, 2003). Although combinatorial chemistry approach has proven successful in optimizing structures of drug compounds, only one *de novo* new chemical entity (NCE) has been approved as a drug [sorafenib (Nexavar) acting as anti-tumor] in these 25 plus years from this method (Cragg and Newman, 2007).

On the other hand, bioactive compounds isolated from natural biological sources offer a vast and unexplored diversity of chemical structures, unmatched by even the biggest combinatorial databases (Strobel and Daisy, 2003). Since thousands of years, natural products have served as traditional medicine and still provide the most affordable treatment for diseases in many developing countries (Amzat and Razum, 2018). Around 40% of modern drugs and 49% of new chemical products registered by the United States Food and Drug Administration (FDA) are based on natural products or their derivatives (Brewer, 2000). Bioactive compounds are naturally derived metabolites and/or by-products from microorganisms, plants, or animals (Baker et al., 2000). Since the past 25 years, bioactive compounds from many traditional medicinal plants have been screened for their antiviral activity by various research groups in Asia, Far East, Europe, and America (Jassim and Naji, 2003).

Particular importance for novel drug discoveries has been bioactive molecules of fungal origin. Especially fungi growing in unique environments such as endophytic and marine fungi are being constantly explored for their antibacterial and antifungal potential. During the past decade, many novel bioactive natural products possessing cytotoxic, anticancer, antibacterial or antifungal activities have been discovered from marine fungi (Mayer et al., 2013; Cheung et al., 2014; Singh et al., 2015). Fungi potentially contain and/or produce several effective molecules that could also be used as antivirals for other hosts. The discovery and characterization of fungal compounds having antiviral activities is an emerging field of research, and several compounds have already been identified as promising. In this review, we go through the present knowledge of fungi-derived extracts and other bioactive agents against viral infection. We especially focus on how the antiviral action has been assessed and how much is known about the mechanisms of action and actual targets.

## Fungi as a Source of Antiviral Agents - an Overview

**FIGURE 1:**
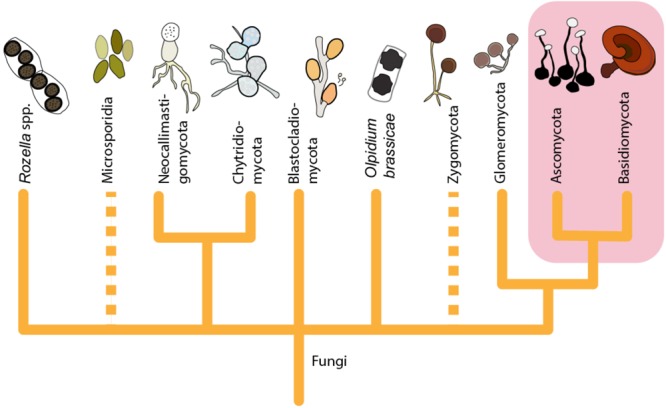
A tree illustrating the larger phylogeny of Fungi shows that the origin of presently known fungal-derived antiviral agents (highlighted) is restricted to the late-diverging fungal phyla (Ascomycota and Basidiomycota). The figure is constructed based on phylogenetic relationships of Fungi on Tree of Life Web Project (http://tolweb.org). This tree is illustrative and does not represent real phylogenetic data. Dashed lines: The group may not be monophyletic, or phylogenetic position of the group is uncertain.

**FIGURE 2:**
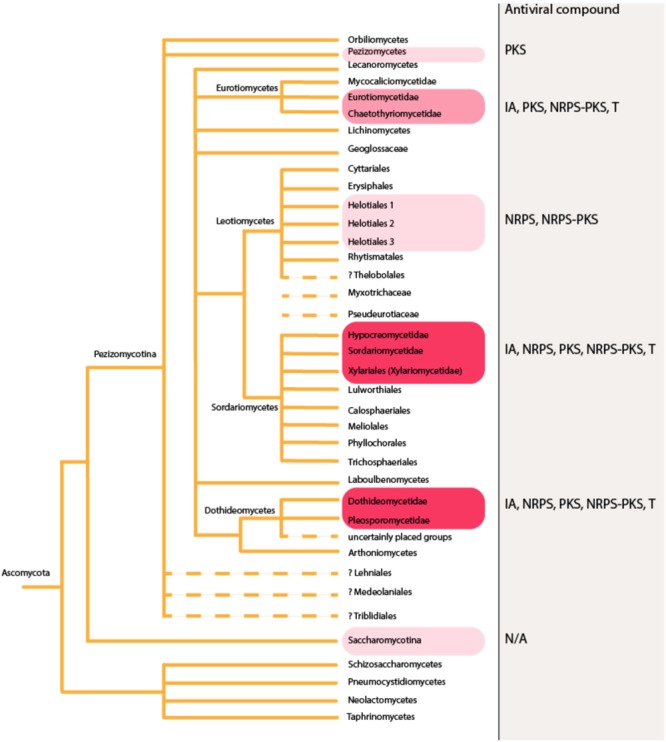
In phylum Ascomycota, antiviral agents have been mainly identified from endophytes and other microfungi restricted to limited number of orders. Higher red color intensity indicates higher number of reports in literature. The figure is constructed based on phylogenetic relationships of Fungi on Tree of Life Web Project (http://tolweb.org). This tree is illustrative and does not represent real phylogenetic data. IA, indole alkaloids; NRPS, non-ribosomal peptides; PKS, polyketides; NRPS-PKS, hybrids; T, terpenoids; N/A, information not available. Dashed lines: The group may not be monophyletic, or phylogenetic position of the group is uncertain.

**FIGURE 3:**
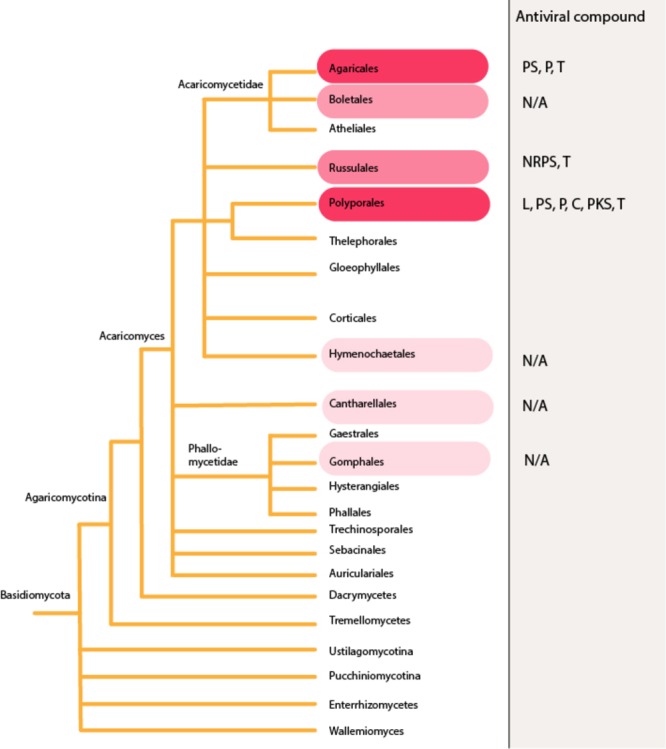
Antiviral agents reported from the phylum Basidiomycota. Higher red color intensity indicates higher number of reports in literature. The figure is constructed based on phylogenetic relationships of Fungi on Tree of Life Web Project (http://tolweb.org). This tree is illustrative and does not represent real phylogenetic data. L, lignin derivative; PS, polysaccharides; P, proteins; C, polysaccharide-protein/amino acid complex; NRPS, non-ribosomal peptides; PKS, polyketides; T, terpenoids; N/A, information not available. Dashed lines: The group may not be monophyletic, or phylogenetic position of the group is uncertain.

The kingdom Fungi represents a rich source of various biologically active compounds. During the past decades, thousands of compounds with diverse biological activities have been recognized and continue to be investigated. Fungal compounds with antiviral activities are less extensively studied, but also number of these investigations is on the increase. We have compiled a list of fungal orders with reported positive antiviral activities (**Table 1**) and also mapped this information on illustrative phylogenetic trees (**Figures 1–3**). Fungal species with reported antiviral activities are given in **Supplementary Table S1**. These demonstrate that the previous studies have focused on the late-diverging fungal phyla (Ascomycota and Basidiomycota) and on rather limited taxonomic groups, while several remaining completely uninvestigated.

**Table 1:** Fungal orders with positive antiviral activities.

Phylum	Order	Virus^∗^	Reference
Ascomycota	Amphisphaeriales	EV71^1^, HIV-I^1^	Li et al., 2008; Wang J. et al., 2014; Jia et al., 2015
	Capnodiales	H1N1^1^	Peng et al., 2013; Wu et al., 2014
	Chaetothyriales	HIV-I^4^	Ondeyka et al., 2003; Mlinaric et al., 2005
	Diaporthales	HIV-1^4^, HSV-1^1^	Jayasuriya et al., 2003; Bunyapaiboonsri et al., 2010
	Dothideales	HSV-1^5^	Isaka et al., 2007
	Eurotiales	EV71^2^, DENV^3^, H1N1^2^, HIV-1^4^, H3N2^2^, JEV^1^, Zika virus^2^	Omura et al., 1993; Matsuzaki et al., 1995; Singh et al., 2003a; Shiomi et al., 2005; Sebastian et al., 2011; Zhang et al., 2011; Gao et al., 2013a; He et al., 2013; Bashyal et al., 2014; Fang et al., 2014; Peng et al., 2014; Wang J.-F. et al., 2014; Stierle and Stierle, 2015; Yu et al., 2016; Raekiansyah et al., 2017
	Glomerellales	HIV-1^4^	Mlinaric et al., 2005
	Helotiales	HSV-1^1^	Rowley et al., 2003
	Hypocreales	EV71^2^, HIV-1^4^, HSV-1^1^, H1N1^1,4^, H3N2^1,4^	Hazuda et al., 1999; Yoshimoto et al., 1999; Minagawa et al., 2002; Singh et al., 2003a,b; Sawadjoon et al., 2004; Mlinaric et al., 2005; Jiang et al., 2011; Ma et al., 2013; Li et al., 2014; Zhao et al., 2017; Pang et al., 2018
	Microascales	HIV-1^4^	Mlinaric et al., 2005
	Ophiostomatales	HIV-1^4^	Mlinaric et al., 2005
	Pezizales	HIV-1^4^	Pérez et al., 2014
	Pleosporales	HIV-1^4^, HSV-1^1^	Hazuda et al., 1999; Singh et al., 2002; Guo et al., 2009; Shushni et al., 2011; Bashyal et al., 2014; Zhang et al., 2015
	Saccharomycetales	HIV-1^4^	Mlinaric et al., 2005
	Sordaliales	HIV-1^4^, influenza A and B^4^	Mlinaric et al., 2005; Sacramento et al., 2015
	Xylariales	H1N1^2^, HIV-1^4^, HSV-1^1^	Hazuda et al., 1999; Pittayakhajonwut et al., 2005; Zhang et al., 2016
Basidiomycota	Agaricales	BoHV-1^1,3^, H1N1^2^, HCV^5^, HBV^4,5^, HCV^5^, HIV-1^2^, HSV-1^1,2,3^, HSV-2^1,2^, influenza A^2^, polio^2^, RSV^1,2^, vaccinia^1^, VS^1^, VZV^2^, WEE^2^	Kandefer-Szerszeń et al., 1980; Amoros et al., 1997; Saboulard et al., 1998; Piraino and Brandt, 1999; Wang and Ng, 2000, 2001; Sorimachi et al., 2001; Lehmann et al., 2003; Chen et al., 2004; Mlinaric et al., 2005; Bruggemann et al., 2006; Grinde et al., 2006; Faccin et al., 2007; Razumov et al., 2010; Zhu et al., 2010; Cardozo et al., 2011, 2014; Gao et al., 2013b; Yamamoto et al., 2013; Krupodorova et al., 2014
	Boletales	HIV-1^4^, HSV-1^5^, vaccinia^1^, VS^1^	Kandefer-Szerszeń et al., 1980; Kanokmedhakul et al., 2003; Mlinaric et al., 2005
	Cantharellales	HIV-1^4^, vaccinia^1^	Kandefer-Szerszeń et al., 1980; Mlinaric et al., 2005
	Gomphales	vaccinia^1^	Kandefer-Szerszeń et al., 1980
	Hymenochaetales	influenza A and B^4^	Ichimura et al., 1998; Awadh Ali et al., 2003;
	Polyporales	BoHV-1^1^, EBV-A^3^, EV71_2_, H1N1^2^, H3N2^2^, HCV^2^, HHV-1^2,4^, HIV^4^, HSV-1^1,2,4^, HSV-2^1,2^, influenza A^2^, MCMV^1,2^, measles^2^, mumps^2^, polio^1,2,3^, PV-1^1^, VSV^2^, WEE^2^, EMCV^2,4^	Hirose et al., 1987; Okada and Minamishima, 1987; Tochikura et al., 1987, 1988; Suzuki et al., 1989; Sorimachi et al., 1990; Sarkar et al., 1993; Amoros et al., 1997; Collins and Ng, 1997; El-Mekkawy et al., 1998; Min et al., 1998; Eo et al., 1999a,b, 2000; Kim et al., 2000; Iwatsuki et al., 2003; Mothana et al., 2003; Ngai and Ng, 2003; Singh et al., 2003a; Mlinaric et al., 2005; Niedermeyer et al., 2005; Gu et al., 2007; El Dine et al., 2008; Sato et al., 2009; Razumov et al., 2010; Rincão et al., 2012; Teplyakova et al., 2012; Krupodorova et al., 2014; Zhang et al., 2014; Matsuhisa et al., 2015; Mizerska-Dudka et al., 2015
	Russulales	HIV-1^4^, vaccinia^1^, VS^1^	Kandefer-Szerszeń et al., 1980; Mlinaric et al., 2005; Wang et al., 2007

*Categories for antiviral methods used in the studies: ^*1*^plague reduction assay; ^*2*^CPE (cytopathic effect) inhibition assay; ^*3*^microscope immunofluorescent assay; ^*4*^Specific protease assay; ^*5*^other. ^∗^WEE, Western equine encaphilitis; VZV, Varicella zoster; RSV, respiratory syncytial virus; HCV, hepatitis C virus; HBV, hepatitis B virus; MCMV, murine cytomegalovirus; VSV, vesicular stomatitis virus; EBV, Epstein-Barr virus; PV-1, poliovirus 1; WNV, west nile virus; HHV, human herpes virus; EMCV, encephalomyocarditis virus; DENV, Dengue Virus; JEV, Japanese encephalitis virus.*

Particularly well-studied for their biologically active compounds, including antivirals, are edible and medicinal mushrooms. Another group of fungi that has been a focus of interest are endophytic fungi, particularly those that grow in marine habitats. The biologically active compounds can be roughly divided into two major groups of molecules; the high-molecular weight compounds present in the extracts and products derived from the fruiting bodies of edible and medicinal mushrooms, and the small organic molecules (secondary metabolites) excreted by the endophytic and other fungi in a liquid culturing (fermentation) setups.

Further rough division can be made when considering the repertoire of antiviral compounds found from different fungal taxonomic groups. Mapping the antiviral compounds on the larger phylogeny of Fungi (**Figure 1**) demonstrates that all the currently known secondary metabolites have been identified from Ascomycota and Basidiomycota. Ascomycota with antiviral activities includes endophytes and other microfungi restricted to limited number of orders (**Figure 2**), while the edible and medicinal mushrooms in the Agaricales and Polyporales (Basidiomycota) (**Figure 3**) are recognized as a source of high-molecular weight compounds. The increasing number of published fungal genome data combined with the traditional bioactivity screening methods has provided novel insights into the true capacity of fungi as producers of bioactive compounds (Bergmann et al., 2007; Khaldi et al., 2010; Brakhage, 2013; Clevanger et al., 2017). These studies indicate that differences exist between these two phyla in a number of secondary metabolites biosynthetic gene clusters and their dominance in their genomes; basidiomycetes typically having fewer compared to ascomycetes (Brakhage, 2013). However, the reported differences between Ascomycota and Basidiomycota reflect also to the bias from the different methods that have been commonly used in screening their biologically active compounds, not differences in their true arsenals of bioactive compounds.

The most recent estimates predicting fungal species diversity indicate that only 3–8% of existing fungal species are discovered and described (Hawksworth and Lücking, 2017). Therefore, the fungi investigated and found to have potential positive antiviral activities thus far represent only a minute fraction of these organisms and their potential.

### Edible and Medicinal Mushrooms

Mushrooms have been an important part of our diet for centuries due to their nutritional properties. Their rich content in proteins, carbohydrates, minerals, vitamins, unsaturated fatty acids and low values of fat and energy content makes them a valuable food source (Barros et al., 2007, 2008; Çağlarırmak, 2007; Kalač, 2009; Ouzouni et al., 2009; Reis et al., 2012).

Some species producing conspicuous fruiting bodies have a long history of medicinal use. Bioactive compounds of the fungal genera which have had an important role in traditional medicine, such as *Ganoderma*, have been subject to extensive research. However, there is a broad number of other edible and medicinal species from different genera considered to be potential antiviral precursors (**Supplementary Table S1** and **Figure 3**). The antiviral activity of these mushrooms is associated mainly to the presence of polysaccharides in mycelium and fruiting bodies, and synthesis of triterpenoid secondary metabolites (Chen et al., 2012; Rincão et al., 2012). However, large number of other potentially bioactive compounds and/or genes involved in their synthesis has been reported (Shiao, 2003; Chen et al., 2012), indicating that the full potential of mushroom and medicinal fungi as a source of bioactive compounds remains only partially understood. Previous study has reported considerable differences in the contents of bioactive compounds produced at different stages of fungal life cycle (Chen et al., 2012), implying that antiviral studies need to take into account the phenotypic variation and growth conditions of the fungal material.

### Endophytes, Marine Fungi and Plant Pathogens

Endophytic fungi that inhabit above-ground tissues of healthy plant at least part of their life cycle are highly diverse in terms of species richness. These primarily ascomycetous (Ascomycota) fungi common in all terrestrial habitats are considered to have important ecological roles in the terrestrial plant communities. Their interactions with host plants and cross-talk with other endophytic microorganisms colonizing the same plant are complex and dynamic (Kusari et al., 2012). Endophytic fungi have been recognized as a rich source of secondary metabolites, which role in the natural habitat likely include chemical signaling, defense against other microorganism, and establishment of symbiosis with host plant (Schulz and Boyle, 2005; Yim et al., 2007; Khaldi et al., 2010). Some also mimic plant defense compounds, and can, therefore, protect host plants against herbivores and pathogens (Kusari et al., 2012). These secondary metabolites are known to have great chemical variety and numerous biological activities with pharmaceutical and biotechnological potential.

It has been hypothesized that extreme habitats harbor greater changes for novel drug discovery (Thatoi et al., 2013; Chávez et al., 2015). Interestingly, rich fungal species diversity inhabits extreme environments such as deep-sea sediments and mangrove ecosystems (Kumaresan and Suryanarayanan, 2001; Mahé et al., 2013). Many of ascomycetous species found in these habitats have been discovered having antiviral and other biological activities (Desmukh et al., 2018). The extreme conditions are thought to shape the secondary metabolite patterns of fungi, and these fungi are recognized as a particularly promising source of diverse and structurally unprecedented novel compounds, which some have already been structurally characterized and several been discovered to constitute of novel carbon skeletons (Saleem et al., 2007).

However, also already relatively well-known fungi should not be overlooked. Less intensively investigated fungi for their bioactivities include tree-pathogens that also seem promising source of antiviral agents. A previous study has detected a number of plant pathogenic fungi with various ecological roles (white-rot fungi, soft-rot fungi, blue-stain fungi and insect-symbionts) having antiviral activities (Mlinaric et al., 2005).

### Antiviral Research and Fungal Taxonomy

Accurate organism identification is the basis for any biological research and its applications. This is particularly important for bioactive compounds aimed for pharmaceutical products. When the physical material used is reported with a misapplied name, the reproducibility of the study is very low. Unfortunately, in the literature on bioactivity and mechanisms of action of isolated compounds or crude extracts of fungal origin, reporting on the methods used to identify fungal materials reveals insensitivity to the relevant taxonomic discussion. Methodologically, only a minority of studies have included a combination of morphological and molecular methods for species identification (Raja et al., 2017). Given the factual diversity of kingdom Fungi, and the resulting difficulties in delimitating species and genera, as well as constant discoveries of species new to science (Hawksworth and Lücking, 2017), transparency in this matter is paramount. Long lasting debates among taxonomists, whether to accept new names, splitting of an old species into many new, or combinations of old names are an everyday affair in the field. This has in some cases resulted in considerable nomenclatural stratification, highlighting the need to engage taxonomists also in the study of applications.

To illustrate this problem, we evaluated literature on one of the most commonly reported name appearing in fungal antiviral research, ‘*Ganoderma lucidum*,’ as well as other species in the genus *Ganoderma* Karst. The poroid, saprotrophic fungal species *G. lucidum* (W. Curt. : Fr.) Karst is an concise example of the broader issue. The traditional medicinal use of *Ganoderma* spp. in East Asia, South-East Asia, and Africa has promoted interest in studying the bioactivity of these fungi, with ‘*G. lucidum*’ often cited as the species of the material. However, exact delimitation of the species concept for *G. lucidum*, with a European type locality, has been difficult due to lack of a holotype specimen (Steyaert, 1972; Moncalvo and Ryvarden, 1997). After morphological and molecular phylogenetic studies on the diversity of the genus in the past decades (Moncalvo et al., 1995; Cao et al., 2012; Zhou et al., 2015), the consensus in the taxonomic literature is that the industrially cultivated “Linghzi” and “Reishi” do not represent the *G. lucidum s. str*, but in fact other species (Wang et al., 2009; Cao et al., 2012). Therefore, careful consideration is required when identifying such samples under this name. Here, we listed the reported methods of acquisition and identification used in each antiviral study on *Ganoderma* (**Supplementary Table S2**). As a summary, out of the 13 studies, only four used material that we can safely assume to represent the species declared, as a fungal taxonomist was being consulted. In eight cases it seems unlikely given the sourcing of the materials, but could in principle be verified to the contrary, assuming access to the original material in herbaria. In one case, the experimental set-up is likely not reproducible due to vague description of the material used, and apparent lack of any preserved specimens. No studies reported sequence data accession numbers, nor morphological criteria used for species determination. Various forms of authorship, including outdated and erroneous, were present with the name *G. lucidum*.

Whether fungal material is in fact correctly identified, has consequences to the independent reproducibility of the study, and reflects also to understanding the species characteristics (i.e., requirements and phenotypic variation in artificial cultivation settings). There is yet a limited amount of comparative work on the differences between species and strains of the composition in the bioactive compounds within *Ganoderma*. The publications available at the moment indicate that differences may be considerable (Welti et al., 2015; Hennicke et al., 2016), though assessments into the extent of occurrence of compounds of interest within the genus is again convoluted by the non-transparent reporting of materials (Richter et al., 2015). In conclusion, given the likelihood of misapplied names in the literature, citing studies not reporting identification criteria as evidence on the antiviral potential of *G. lucidum s. str.* needs to take this ambiguity into account.

The misidentification of species and even genera is even more likely with microscopic fungi (such as endophytes) containing minute and overlapping morphological characteristics, and of which taxonomy and diversity remains widely uninvestigated. Therefore, we highlight the importance for transparency in reporting of used nomenclature, physical fungal material and method of identification, which is paramount to the advancement of research on antivirals from fungi. Furthermore, we encourage the natural product research community adopting the recently suggested set of standardized procedures for the identification of fungi (Raja et al., 2017).

### Overview of Methods Assessing Antiviral Activity

The most widely used methods for the initial screening of fungal extracts to evaluate their antiviral activity are the plaque reduction assay (Zhu et al., 2004; Faccin et al., 2007; Rincão et al., 2012), cytopathic effect (CPE) assay (Liu et al., 2004; Zhang et al., 2011) and immunofluorescence assay (Faccin et al., 2007) (**Table 2**). In addition, various commercially available viability assays monitoring for, e.g., the cellular ATP levels have also been used. These assays are also used for performing the time of addition studies and investigating the direct virucidal activity of the fungal extracts (Liu et al., 2004; Faccin et al., 2007).

**Table 2:** Methods used to evaluate antiviral effects.

To study	Method	Read out	Reference
Antiviral activity, Virucidal activity and	CPE assay using crystal violet to stain viable cells	OD values at 550–595 nm	Schmidtke et al., 2001
Time of addition studies	Plaque reduction assay	No of plaques per well-> PFU/ml	Rincão et al., 2012
	Microscopy immunofluorescent assay to label newly synthetized capsid proteins	% of infected cells with respect to untreated infected cells	Faccin et al., 2007
Direct effect on virus	Negative staining TEM	Unstained, intact viruses vs. darkly stained, empty viruses	Myllynen et al., 2016
	Structural studies (e.g., x-ray crystallography or cryo-EM)	Atomistical model exhibiting drug binding or virus opening	De Colibus et al., 2014
	Real-time spectroscopy using SYBR-Green	Fluorescence intensity increase upon genome release	Myllynen et al., 2016
	Density gradient of radioactively labeled virus showing intact and uncoated viruses	Radioactive counts (CPM) per each gradient fraction showing peaks of intact and empty viruses	Marjomäki et al., 2002; Myllynen et al., 2016
Adsorption/receptor attachment	Binding assay	Radioactive counts (CPM) per each gradient fraction showing peaks of intact and empty viruses	Marjomäki et al., 2002; Myllynen et al., 2016
	Computational simulations (molecular docking)	Binding energy upon drug binding (-kcal/mol)	Zhang et al., 2014
Uncoating	Density gradient of radioactively labeled virus showing intact and uncoated viruses	Radioactive counts (CPM) per each gradient fraction showing peaks of intact and empty viruses	Marjomäki et al., 2002; Myllynen et al., 2016
	Real-time spectroscopy Structural studies (e.g., x-ray crystallography or cryo-EM)	Fluorescence intensity increase upon genome release	Myllynen et al., 2016 Hewat and Blaas, 2004; Levy et al., 2010
Replication intermediates (replication) and capsid protein production (translation)	Immunolabeling and confocal microscopy	Fluorescence intensity quantification of capsid or dsRNA production	Martikainen et al., 2015
Specific viral proteases	HIV-1 protease peptide cleavage assay	Monitoring the fluorescence of the enzyme catalyzed reaction	Singh et al., 2004
	X-ray crystallography	Atomistic details for binding	Singh et al., 2004
	scintillation proximity assay (SPA)	Measuring radioactivity of the enzymatic reaction using radioactive biotinylated substrate and streptavidin tagged scintillant	Guo et al., 2000

All of these methods calculate in different ways the viability of the cells after virus action, and the antiviral activity is monitored as the rescue of the cells from the viral infection. The read out for the plaque reduction assay is the visual counting of the number of plaques formed [plaques forming unit (PFU)/ml] i.e., number of unstained “holes” in the culture plate after crystal violet staining of the cells that still adhere on the plate. This number is then used to calculate the percentage of viral inhibition (% V.I.) (Zhu et al., 2004). In immunofluorescence assay, the cells are observed under microscope and typically several hundreds of cells are scored. First, the number of infected cells is calculated from the number of cells showing high abundance of viral capsid proteins produced in the cell cytoplasm (Marjomäki et al., 2002). Then, from the obtained number, V.I. is calculated with respect to untreated infected cells (Faccin et al., 2007). In the case of CPE assay, the read out is based on the spectrophotometric absorbance reading of the stained viable cells, which is used to calculate the % V.I. (Liu et al., 2004). Typically, the viable cells left on the bottom of the culture plate and stained with crystal violet, are dissolved in the lysis buffer to provide a homogenous blue suspension that is easy to measure in the spectrophotometer (Schmidtke et al., 2001). The linear regression analysis of the plots of % V.I. is used to determine the 50% inhibitory concentration (IC_50_) which is used further to calculate the selectivity index (SI) (Rincão et al., 2012). The calculations are also given here as formulas:

% VI calculated from the plaque reduction assay read-out=[1−(number of plaques in test/number of plaques in virus control)]×100 (Rincão et al., 2012)% VI calculated from the CPE assay read-out= [(ODt)v - (ODc)v]/[(ODc)mock - (ODc)v] × 100 (Liu et al., 2004)

where (OD_t_)_v_ is the optical density (OD) of the cell, treated with virus and bioextract (test), (OD_c_)_v_ is the OD of the cell, treated with virus (virus control) and (OD_c_)_mock_ is the OD of the mock infected cell (cell control).

SI = CC50/IC50

Where CC50 is 50% cytotoxic concentration, i.e., the concentration which caused a 50% reduction in the number of viable cells or in the optical density and IC50 is 50% inhibitory concentration, i.e., the concentration capable of reducing 50% PFU in relation to the controls.

These above-mentioned methods only affirm the antiviral potential of bioactive compounds and do not reveal any information regarding their mechanism of action. Only few papers have progressed to evaluate the actual molecular targets. In order to study various viral or cellular targets of drug action, several approaches could be used. To study the direct effect on the virus, there are several methods that could be employed. First of all, perhaps the easiest way to see gross effect on the virus particle is to negatively stain the virus samples and observe them under transmission electron microscope (TEM) (Myllynen et al., 2016). There is a characteristic feature to distinguish between intact viruses from empty particles in TEM imaging. The staining dye, e.g., 2% Uranyl acetate or 1% phosphotungstic acid cannot enter the capsid of intact viruses because of which the intact particles appear bright, i.e., unstained, in TEM images (due to the contrast of the dye). However, in case of empty viruses, since the capsid is open, the dye enters the capsid and stains the insides of the virus thus giving a dark appearance for empty virus particles in TEM images. Density gradient centrifugation of either radioactively labeled or non-labeled virus is also insightful in revealing the direct effect of the extract on the virus (Marjomäki et al., 2002; Myllynen et al., 2016). The read out of radioactive gradient fractionation is the radioactivity [counts per minute (CPM)] of various fractions from different densities showing peaks of more dense intact virus and less dense empty viruses or even smaller products like pentamers. Direct effects of bioactive agents should show clear changes in the fraction of intact versus empty viruses.

The effect on the virus attachment on cellular receptors has been studied using binding assays. Binding is most sensitively studied using radioactively labeled virus and by performing binding assays in cold, hence eliminating the virus entry inside the cells by endocytosis (Marjomäki et al., 2002). Specific effects of molecules interfering with receptor binding have been also performed *in silico* by using molecular docking studies (Zhang et al., 2014). Whether the drug targets the virus uncoating *in vitro* or while the virus is inside cellular compartments, can be evaluated using real-time spectroscopy by using RNA/DNA binding fluorescent dyes (Myllynen et al., 2016) and using radioactive gradient fractionation studies, respectively. Radioactively labeled virus may be isolated from the cells for gradient fractionation which may reveal if there is a block in the viral genome release, thus leaving the virus as intact for longer periods. In order to assess the effect of bioextract on the efficiency of replication and viral translation, immunofluorescent labeling may be performed that reveals production of virus capsid proteins and specific replication intermediates, such as, e.g., dsRNA (Martikainen et al., 2015). Furthermore, qPCR to reveal new viral RNA production may be used.

There are also more specific assays that have been used to test the bioactive agents against virus-specific proteins such as proteases. Those assays have been developed directly against specific viruses such as HIV or human cytomegalovirus (hCMV) (Guo et al., 2000; Singh et al., 2004). Those assays are usually *in vitro* assays relying on purified proteases.

## Bioactive Agents Acting as Antivirals

Small-molecule fungal secondary metabolites have been a source of various drugs, and the same classes of secondary metabolites seem promising also against viruses (**Table 3**). Other bioactive compounds with potential antiviral activities include high molecular weight compounds, such as polysaccharides, proteins and lignin-derivatives.

**Table 3:** Fungal bioactive agents with reported antiviral activities.

Chemical class	Source (fungal order)	Phylum	Reference
**High molecular weight compounds**			
Lignin derivatives	Polyporales	Basidiomycota	Suzuki et al., 1989; Sorimachi et al., 1990; Sarkar et al., 1993
Polysaccharides	Agaricales, Polyporales	Basidiomycota	Faccin et al., 2007; Razumov et al., 2010; Cardozo et al., 2011; Yamamoto et al., 2013
Proteins	Agaricales, Polyporales	Basidiomycota	Piraino and Brandt, 1999; Wang and Ng, 2000; Ngai and Ng, 2003; Gu et al., 2007
Polysaccharide-protein/amino acid complex	Polyporales	Basidiomycota	Hirose et al., 1987; Okada and Minamishima, 1987; Tochikura et al., 1988; Collins and Ng, 1997; Eo et al., 1999a,b, 2000; Kim et al., 2000; Wang and Ng, 2000
**Small molecular weight compounds (secondary metabolites)**			
Indole alkaloids	*Capnodiales, Eurotiales, Hypocreales, Pleosporales*	*Ascomycota*	Guo et al., 2009; Zhang et al., 2011; Ma et al., 2013; Peng et al., 2013; Li et al., 2014; Zhao et al., 2017
Non-ribosomal peptides (NRPS)	*Dothideales, Helotiales, Xylariales*	*Ascomycota*	Rowley et al., 2003; Pittayakhajonwut et al., 2005; Isaka et al., 2007
	*Russulales*	*Basidiomycota*	Wang et al., 2007
Polyketides (PKS)	*Amphisphaeriales, Diaporthales, Eurotiales, Hypocreales, Pezizales, Pleosporales, Sordaliales*	*Ascomycota*	Singh et al., 2002, 2003a; Jayasuriya et al., 2003; Li et al., 2008; Bunyapaiboonsri et al., 2010; Shushni et al., 2011; Gao et al., 2013a; Bashyal et al., 2014; Peng et al., 2014; Pérez et al., 2014, Wang J.-F. et al., 2014; Jia et al., 2015; Sacramento et al., 2015; Pang et al., 2018
	*Polyporales*	*Basidiomycota*	Awadh Ali et al., 2003; Singh et al., 2003a
NRPS-PKS hybrids	*Capnodiales, Eurotiales, Helotiales, Hypocreales, Pleosporales*	*Ascomycota*	Krohn et al., 1997; Hazuda et al., 1999; Bunyapaiboonsri et al., 2010; Sebastian et al., 2011; Wu et al., 2014; Stierle and Stierle, 2015; Zhang et al., 2015
Terpenoids	*Amphisphaeriales, Eurotiales, Hypocreales, Pleosporales, Xylariales*	*Ascomycota*	Hazuda et al., 1999; Yoshimoto et al., 1999; Minagawa et al., 2002; Sawadjoon et al., 2004; Singh et al., 2003b; Fang et al., 2014; Wang J. et al., 2014; Zhang et al., 2014
	*Agaricales, Polyporales, Russulales*	*Basidiomycota*	El-Mekkawy et al., 1998; Min et al., 1998; Iwatsuki et al., 2003; Kanokmedhakul et al., 2003; Krawczyk et al., 2003; Lehmann et al., 2003; Mothana et al., 2003; Niedermeyer et al., 2005; El Dine et al., 2008; Sato et al., 2009; Zhu et al., 2010; Zhang et al., 2016

### Small Organic Molecules (Secondary Metabolites)

Fungal secondary metabolites are low-molecular-weight compounds, which in contrast to primary metabolites, are not directly required for the growth of the organism. Their ecological function in nature remains widely unknown. It has been hypothesized that secondary metabolites contribute to chemical communication with and competition against other organisms (Yim et al., 2007; Khaldi et al., 2010). Some also mimic plant defense compounds, and can, therefore, protect host plants against herbivores and pathogens (Kusari et al., 2012). A majority of known secondary metabolites have been identified from ascomycetes in traditional culture-based screening approaches, particular interest have been marine and plant endophytic fungi (Strobel and Daisy, 2003; Saleem et al., 2007; Kusari et al., 2012; Thatoi et al., 2013; Stierle and Stierle, 2015; Desmukh et al., 2018).

The production of secondary metabolites has been most commonly studied in *in vitro* setups, where the compounds secreted by hyphal cells to a culture medium are studied. Although these studies have formed an important basis for the discovery of fungal bioactive metabolites, it is likely that the true potential of fungi as producers of secondary metabolites has been underestimated. Genome-based projects have provided novel insights and demonstrated that many cryptic gene clusters involved in secondary metabolite biosynthesis are silent or not well expressed in standard cultivation/fermentation conditions traditionally used for screening for the secondary metabolites (Bergmann et al., 2007; Khaldi et al., 2010; Brakhage, 2013; Clevanger et al., 2017). To activate silent biosynthetic gene clusters, altering the growth conditions (such as carbon and nitrogen sources, temperature, light, pH and aeration) have been used as stimuli. However, regulation of secondary metabolism biosynthesis pathway is complex, and these are not universally relevant stimuli for most gene clusters and fungal species (Brakhage, 2013). Genome mining provides novel possibilities for understanding the genetic basis of secondary metabolite production and developing strategies for activation of the silent metabolic pathways (Bergmann et al., 2007; Andersen et al., 2012; Ochi and Hosaka, 2013).

The genome of each fungus contains a remarkable capacity of biosynthetic gene clusters encoding the production of diverse secondary metabolites (Khaldi et al., 2010; Chen et al., 2012; Inglis et al., 2013; Han et al., 2016). Although the secondary metabolites are structurally highly diverse, they are produced by a few common biosynthetic pathways (Keller et al., 2005). Previous studies which have applied genome predictions have identified non-ribosomal peptide synthase (NRPSs) and polyketide synthase (PKSs) gene clusters being the most abundant, while also hybrid NRPS-PKS enzymes, prenyltransferases (DMATSs), terpene cyclases (TCs) are commonly present in fungi (Bergmann et al., 2007; Khaldi et al., 2010; Andersen et al., 2012; Han et al., 2016). These “backbone” enzymes are responsible for the synthesis of the secondary metabolite core structures which include non-ribosomal peptides, polyketides, NRPS-PKS hybrids, indole alkaloids and terpenoids, respectively (Hoffmeister and Keller, 2007). The synthesized core structures and product intermediates are typically further modified by tailoring enzymes before the final product is transported outside the fungal cell (Andersen et al., 2012; Brakhage, 2013).

## High Molecular Weight Compounds

The fungal cell wall is an essential structure component that protects the cells against the environment and other organisms. The fungal cell wall allows the selective exchange of compounds with other cells and with their surroundings. Apart from that, it also provides of shape and strength to the fungal cell. The composition of the cell wall varies between fungal species and within the same species or strains (e.g., growth stage, growth conditions, environmental factors). Despite the variability of the composition, the main components that can be commonly found in mushrooms are proteins and polysaccharides (Bowman and Free, 2006). High molecular weight polysaccharides (such as glucan, chitin, mannan, PSK or lentinan) extracted from fruiting bodies and fungal mycelia have been reported to present antiviral activities (Tochikura et al., 1987, 1988; Cardozo et al., 2011; Rincão et al., 2012).

To study the mechanism of action of polysaccharides against a determinate virus, its chemical composition must be understood. The analysis of the structure of polysaccharides is a complex task that requires several isolation steps. When polysaccharides are extracted from a fungal sample, the determination of the purity becomes a priority in order to understand the chemical structure. Knowledge on the monosaccharide composition, the linkage positions between glycosidic linkages, the distinction of furanosidic and pyranosidic rings, the anomeric configuration, the sequences of monosaccharide residues and repeating units, the substitutions and the molecular weight including its distribution are essential to define the structure of a certain polysaccharide (Cui, 2005).

The presence of proteins in the cell wall has also a protective function since they are the responsible of stimuli perception (Geoghegan et al., 2017). Hence, proteins are involved in the production and regulation of secondary metabolites (Bok and Keller, 2016). Moreover, their presence in the cell wall in combination with polysaccharide complexes allows the interaction with the environment, helping to the fungal cell to the transport of substances in and out of the fungal cell.

Several reports refer to replication inhibition for several virus types, suggesting that both polysaccharides and proteins act at the first phases of viral replication system (Tochikura et al., 1988; Collins and Ng, 1997; Eo et al., 1999a,b, 2000; Piraino and Brandt, 1999; Kim et al., 2000; Wang and Ng, 2000; Ngai and Ng, 2003; Gu et al., 2007; Cardozo et al., 2011; Yamamoto et al., 2013). However, the interaction of proteins and polysaccharides with the viral replication system is not completely understood.

## Antiviral Mechanisms

### Possibility to Act at Different Stages of Virus Life Cycle

**FIGURE 4:**
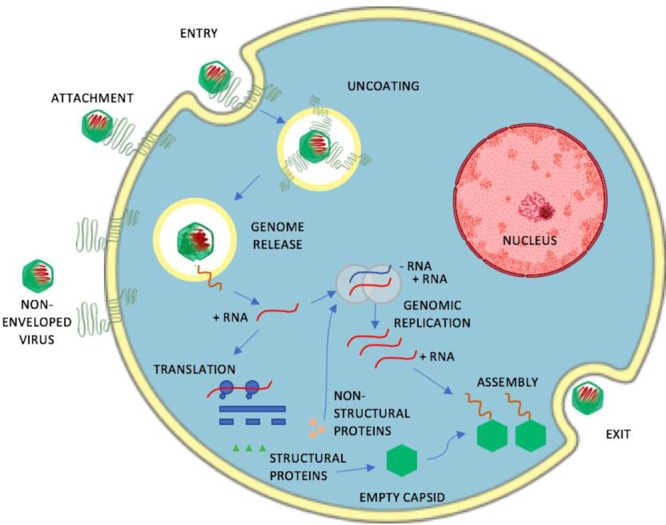
A schematic representation of the life-cycle of a non-enveloped, positive sense single-stranded RNA. The infection stages of the virus that include attachment, entry, uncoating, genome release, genomic replication, translation, assembly and exit serve as potential targets for antivirals.

There are several possibilities to interfere viral infection (**Figure 4**). Viruses can be directly attacked outside cells in order to destroy the viral particles before their attachment on cellular receptors. Such agents could irreversibly modify viral particles on different surfaces, or, if being non-toxic, also in human body. For human enteroviruses, several molecules have been designed to fit in to the special hydrophobic pocket, thus replacing the aliphatic fatty acid normally housed in the virus particle (De Colibus et al., 2014). The hope in this strategy is to stabilize the virus particle and prevent virus uncoating. This pocket is also close to the receptor binding area and thus molecules targeted to the pocket could potentially inhibit receptor binding. These molecules have shown some potency in their antiviral effect. However, binding to these pockets is usually dynamic, and the effects in long-term studies have not been successful. However, during short time periods, these molecules have shown efficacy.

Inhibiting the receptor binding is another possibility to prevent virus infection. When several virus groups use similar receptors, this strategy offers a nice possibility to prevent viral infection, e.g., in the case of heparan sulfate binding (Cagno et al., 2018). Most viruses use cytoplasmic endosomes as their portal for cellular entry. This may be considered as a true rate limiting step for many viruses, especially those of non-enveloped viruses. By using fusogenic peptides that normally act in low pH, enveloped viruses have developed means to fuse with the limiting membrane of the endosomes, thus releasing their contents to the cytoplasm. Thus, several molecules that prevent the lowering of the endosomal pH quite effectively inhibit viral infection. However, as the low pH is such a crucial event for normal nutrient uptake and signaling through endosomes, such strategies are not really feasible. Non-enveloped viruses do not usually rely on low acidity. We have shown recently that enteroviruses in general do not use low pH in their strategy to infect cells, but rather accumulate in endosomes with higher acidity (Marjomäki et al., 2015). There is very little information yet available on the strategies of non-enveloped viruses to break the endosomal barrier.

RNA-based viruses start their translation and transcription usually in the cytoplasm. Those events are a good target for several antivirals as many of the mechanisms could target a larger amount of virus groups. DNA-viruses travel to the nucleus and start their replication there. The replication for several viruses take advantage of various membranous organelles as usually the replication occurs on the surface of membranous structures. Indeed, virus infection usually strongly perturbs the functioning of various endosomes, ER and Golgi for the profit of viruses. In addition, often cholesterol and some more rare forms of lipids are being utilized for viral replication, and may serve as targets for combatting viral infection.

During translation and replication, in addition to the viral structural proteins, several non-structural viral proteins are being synthetized in the cytoplasm. Several present strategies against viral infection target the viral proteases or viral polymerases and may prove successful in preventing viral infection.

Virus assembly occurs in the cytoplasm for several non-enveloped viruses that, in the end, causes lytic disruption of the cell and spread of the progeny viruses to neighboring cells. The lytic events are often preceded by activation of caspases to promote apoptosis. Viruses are master manipulators of anti-apoptotic growth factor signaling and proapoptotic caspase pathways. Usually viruses try to prevent apoptosis during the early infection but may boost apoptotic processes later to facilitate an efficient spread to the cell surroundings. Therefore, the strategies to manipulate apoptosis may be complicated. However, we showed previously that targeting BCl-molecules, thus boosting apoptosis, facilitated killing of virus infection early, and prevented possibilities for influenza and HSV to develop difficult symptoms usually encountered with virus infection (Bulanova et al., 2017). Thus, maybe in combination with other antivirals, this strategy could perhaps be used for antiviral action.

Enveloped viruses take their envelope usually from the plasma membrane and use some components of the cellular machinery, ESCRTS, to facilitate the topologically outbound formation of viral particles, as in the case of HIV viruses budding from cells. Attacking those ESCRT components could potentially prevent virus spread but also would target the elementary aspects of multivesicular structure formation of endosomes.

### Mechanisms Found So Far

For mechanistic studies, rather limited numbers of viruses have been studied so far. Herpes simplex virus (HSV) has been most thoroughly tested against some purified and unpurified fungal products (**Table 4**). In addition to HSV, also influenza viruses (IF) have been tested against some purified fungal products. Both HSV and IF are enveloped viruses that are in general suspected to be more prone to degradation and destabilization. In contrast, non-enveloped viruses are considered more robust and may keep stabile even in the harsh conditions for long time periods. Maybe therefore, less hits have been discovered from fungal products. However, poliovirus, a member of non-enveloped enteroviruses, has been shown to be affected by *Lentinula edodes* and *Agaricus subrufescens* -derived products (**Table 4**). In addition, triterpenoids from *G. lucidum* have been shown to effectively reduce the infectivity of enterovirus 71 (**Table 4**).

**Table 4:** Antiviral mechanisms.

Mechanism	Source^∗^	Bioactive agent	Efficacy	Target virus	Reference
Directly on virus particle	*Agaricus subrufescens*^a^	N/A	50 μg/ml; %VI = 78.4 ± 0.16	HSV-1	Bruggemann et al., 2006
			1000 μg/ml; %VI = 73.9 ± 0.38		
			50 μg/ml; %VI = 51 ± 0.33	BoHV-1	
			1000 μg/ml; %VI = 46.8 ± 0.16		
	*Grifola frondosa*^b^	GFAHP	IC50 (μg/ml) -4.10 ± 1.50	HSV	Gu et al., 2007
			TI50 (CC50/IC50) ->120		
Adsorption	*Agaricus subrufescens^a^*	Beta-glucan-protein	800 μg/ml; %VI = 77.5	HSV	Yamamoto et al., 2013
	*Agaricus subrufescens*^a^	Beta-glucan-protein	EC50 - 0.32 ± 0.05	HSV-1 (KOS)	Cardozo et al., 2014
			EC50 - 0.10 ± 0.4	HSV-2 (333=	
			EC50 - 2.07 ± 0.06	HSV 1 (gc 39)	
			EC50 - 0.51 ± 0.13	HSV-2 (gCneg1)	
	*Chaetomium coarctatum*	Aurenitol	EC50 (nM) - 100 ± 16	A (H3N2)^∗^	Sacramento et al., 2015
			EC50 (nM) -300 ± 23	A (H3N2)	
	*Lentinula edodes*	N/A	Aq. Ex. IC50 (mg/ml)-12.7; SI-5.82	PV	Rincão et al., 2012
			EtOH Ex. IC50 (mg/ml)-1.3; SI-19.85		
			PLS. Ex. IC50 (mg/ml)-0.19; SI > 21.33		
			Aq. Ex. IC50 (mg/ml)-8.2; SI-9.02	BoHN-1	
			EtOH Ex. IC50 (mg/ml)-2.13; SI-12.11		
			PLS. Ex. IC50 (mg/ml)-0.1; SI > 39.21		
	*Trametes versicolor*^b^	Polysaccharopeptide	1.5 μM; IC50 - 0.15 mg/ml	HIV	Collins and Ng, 1997
Replication	*Agaricus subrufescens*^a^	Polysaccharides	IC50 – 97.2; SI – 9.9	PV	Faccin et al., 2007
	*Trametes versicolor*	N/A	EC50 - 0.077 mg/ml; TI - 324.67	Influenza, HSV	Krupodorova et al., 2014
Virus proteins as targets	*Cordyceps militaris*	Adenosine	No quantifiable results	HIV protease	Jiang et al., 2011
	*Flammulina velutipes*^a^	Velutin	5 mg/ml; %VI = 94.8 ± 5.6	HIV reverse transcriptase	Wang and Ng, 2001
	*Russula paludosa*	4.5 kDa protein	IC50-0.25 mg/ml	HIV protease	Wang et al., 2007
	*Ganoderma lucidum*^a,b^	Ganoderic acid	GLTA Binding energy(-kcal/mol)-11.95	HIV protease	Min et al., 1998
	*Ganoderma lucidum*^a,b^	Triterpenoids	GLTA Binding energy(-kcal/mol)-13.07	EV71	Zhang et al., 2014
	*Ganoderma sinense*^b^	Triterpenoids	IC50 -20-40 μM	HIV protease	Sato et al., 2009

*^∗^Species names used in the table have been amended to the current accepted name for the taxon declared in the cited studies. In cases where the declared name has since been revised in such a way as to make the conspecificity of the material ambiguous, the name is followed by ^*a*^. If there are no identification methods reported in cited original studies, the name is followed by ^*b*^. VI, viral Inhibition; TI, therapeutic index; IC, inhibitory concentration; EC, effective concentration; ^∗^lab strain: Aq. Ex., aqueous extract; Si, selectivity index; EtOH Ex, ethanol extract; PLS Ex, polysaccharide extract; GLTA, Lanosta-7,9(11),24-trien-3-one,15;26-dihydroxy; GLTB, ganoderic acid Y*.

There are several published antiviral studies especially with edible mushrooms and with their aqueous and ethanol/methanol extracts. Most studies on antiviral action have been performed using standard plaque assay or CPE assay, measuring the amount of infective particles after the treatment. With such assays, the inhibitory action may have occurred during any step of the viral infection, starting from direct action on the virus particle itself. More information on the inhibitory effect has been acquired from time of addition studies, where inhibitory molecules were added at different stages along viral infection (Faccin et al., 2007; Yamamoto et al., 2013). These studies have pinpointed several extracts and isolated molecules that showed inhibitory action directly on the virus particles or on the adsorption of the virus on cells (see **Table 4**). More detailed studies with *Ganoderma* triterpenoids using molecular docking tools showed affinity to the hydrophobic pocket of enterovirus 71 suggesting that either uncoating or binding to the cellular receptor could be affected (Zhang et al., 2014). These triterpenoids showed best efficacy when they were first mixed with the virus before adding on cells, confirming that either uncoating or binding on cells indeed were targeted. However, without further analysis addressing those steps with specific binding assays or uncoating assays the actual mechanism remains unknown.

Some studies showed preferential inhibition still some hours p.i. suggesting that the inhibitory action was probably in the viral protein translation or replication. More direct studies have been done with assays that specifically target viral proteins *in vitro*. These studies have been performed most heavily with HIV proteases and reverse transcriptases. Such studies have pinpointed ganoderic acid and triterpenoids, as well as adenosine, velutin and a novel 4.5 kDa agent to directly act on HIV proteins (Sato et al., 2009). In many cases the bioactive compound is chemically modified to increase its antiviral potency. Cardozo et al. (2011), produced a sulfated derivative of a polysaccharide, isolated from *Agaricus brasiliensis* and found that the sulfated polysaccharide showed increased antiviral activity against HSV I.

## Future Perspectives

Currently, numerous fungal-derived metabolites such as lovastatins, antibiotics and antifungal agent griseofulvin are present on the drug markets. Fungal-derived compounds have not been approved for antiviral treatment. However, as numerous previous studies have found many of them exhibiting potential antiviral efficacy agents (**Tables 1, 4**), it is probably only a matter of time before some molecules will be taken for clinical testing. The effective antiviral fungal compounds showing the best ADME (pharmacokinetic characteristics adsorption, distribution, metabolism and excretion) *in vitro* will be taken for animal testing *in vivo*. However, thus far there are very few well-designed, high-quality clinical trials on treatments with fungal-derived standardized pharmaceuticals (Zhou et al., 2005; Gargano et al., 2017).

Standardizing the biosynthesis of biologically active compounds for trials, as well as up-scaling to industrial scale has to deal with complexity of fungal biology and ecology. The interspecies interactions are known to influence the fungal metabolism in the organism’s native environment, but their importance in biotechnological applications remains an underexplored issue (Kusari et al., 2014a,b). Intraspecies genetic and morphological variation complicates the optimization of cultivation conditions (Posch et al., 2013). Still, increasing number of fungal genome sequences in combination with metabolomics provide novel possibilities for understanding the regulation of secondary metabolism, enhancing the yields of target compounds, as well as providing a platform for novel drug discoveries (Harvey et al., 2015).

Alongside liquid cultivation, potential fungal antivirals have been extracted from harvested sporocarps, especially in studies on basidiomycetes. Domestication of various such sporocarp-producing species has been successful within industrial symbiosis built on easily obtainable lignocellulosic waste from agriculture and forestry. This approach has been supported in some cases by the observed difficulties in obtaining particular metabolites otherwise (Chen et al., 2012), though there are known issues of economic costs and quality control (Hu et al., 2017; Wu et al., 2017).

Despite these numerous challenges with species that have been studied in some cases for decades, it is also important to continue investigating fungal species diversity, as only a small number of the known fungi have been investigated for antiviral activity. Whereas biodiversity hot-spots and little-explored habitats are particularly important for finding unrecognized fungal species, cryptic species represent considerable genetic reserve also in long studied ecosystems (Hawksworth and Lücking, 2017). Multidisciplinary engagement between virologists and fungal taxonomists is particularly pressing in this case.

## Conclusion

As fungi are a rich source of bioactive agents, the accumulation of know how on the actual bioactive molecules enriched, and their detailed targets in virus families will probably increase in the near future. Presently, there is a rather limited understanding of the antiviral mechanisms of fungal products on virus infection. Thus, more detailed knowledge on the actual molecular targets is crucial in order to develop these molecules further to efficiently combat virus infections in the future. Laboratory assays targeting directly various steps along virus infection are needed to understand in detail the mechanisms of action.

## Author Contributions

All authors actively taken part in developing the idea of this review article. The literature review and manuscript writing were performed by RL, DR, PV, MC-E, and VM. HV arranged the required resources and commented on the manuscript. All authors contributed to the finalizing of the manuscript and approved it for publication.

## Conflict of Interest Statement

The authors declare that the research was conducted in the absence of any commercial or financial relationships that could be construed as a potential conflict of interest.
