# Supplementary material for: Antiviral Agents From Fungi: Diversity, Mechanisms and Potential Applications
Source: Front Microbiol. 2018 Oct 2;9:2325. doi: 10.3389/fmicb.2018.02325 (PMC6176074; doi:10.3389/fmicb.2018.02325)
Supplement: Supplementary file 1 [file Table_1.docx]

**Supplementary table 1.** Fungal species with positive antiviral activities.

| Fungal species* | Order | Phylum | Reference |
| --- | --- | --- | --- |
| *Agaricus subrufescens ^a^* | Agaricales | Basidiomycota | Sorimachi et al., 2001; Chen et. al. 2004; Bruggemann et al., 2006, Grinde et al., 2006, Faccin et al., 2007; Cardozo et al., 2011, 2014; Yamamoto et al., 2013 |
|  |  |  |  |
| *Agrocybe salicacola* | Agaricales | Basidiomycota | Zhu et al., 2010 |
| *Alternaria tenuissima* | Pleosporales | Ascomycota | Bashayal et al., 2014 |
| *Amanita pantherina ^c^* | Agaricales | Basidiomycota | Kandefer-Szerszeń et al., 1980 |
| *Amanita spissa ^c^* | Agaricales | Basidiomycota | Kandefer-Szerszeń et al., 1980 |
| *Fibroporia vaillantii ^c^* | Polyporales | Basidiomycota | Mlinaric et al., 2005 |
| *Armillaria mellea ^c^* | Agaricales | Basidiomycota | Kandefer-Szerszeń et al., 1980 |
| *Ascochyta* sp. *^c^* | Pleosporales | Ascomycota | Singh et al., 2002 |
| *Aspergillus ochraeus ^a^* | Eurotiales | Ascomycota | Fang et al., 2014 |
| *Aspergillus sydowii ^a^* | Eurotiales | Ascomycota | Wang et al., 2014b |
| *Aspergillus terreus ^a,c^* | Eurotiales | Ascomycota | Gao et al., 2013, He et al., 2013 |
| *Auriporia aurea ^c^* | Polyporales | Basidiomycota | Krupodorova et al., 2014 |
| *Bjerkandera adusta ^c^* | Polyporales | Basidiomycota | Mlinaric et al., 2005 |
| *Boletus edulis ^c^* | Boletales | Basidiomycota | Kandefer-Szerszeń et al., 1980 |
| *Cantharellus cibarius ^c^* | Cantharellales | Basidiomycota | Kandefer-Szerszeń et al., 1980 |
| *Ceratocystis coerulescens ^a,c^* | Microascales | Ascomycota | Mlinaric et al., 2005 |
| *Cerrena unicolor* | Polyporales | Basidiomycota | Mizerska-Dudka et al., 2015 |
| *Chaetomium coarctatum ^a,c^* | Sordaliales | Ascomycota | Sacramento et al., 2015 |
| *Chaetomium globosum ^a,c^* | Sordaliales | Ascomycota | Mlinaric et al., 2005 |
| *Chondrostereum purpureum ^c^* | Agaricales | Basidiomycota | Mlinaric et al., 2005 |
| *Cladosporium sphaerospermum ^a,c^* | Capnodiales | Ascomycota | Wu et al., 2014 |
| *Cladosporium* sp. | Capnodiales | Ascomycota | Peng et al., 2013 |
| *Clitocybe nebularis ^c^* | Agaricales | Basidiomycota | Kandefer-Szerszeń et al., 1980 |
| *Rhodocollybia butyraceae ^c^* | Agaricales | Basidiomycota | Kandefer-Szerszeń et al., 1980 |
| *Gymnopus dryophilus ^c^* | Agaricales | Basidiomycota | Kandefer-Szerszeń et al., 1980 |
| *Rhodocollybia maculate ^a,c^* | Agaricales | Basidiomycota | Amoros et al., 1997 |
| *Coniophora puteana ^c^* | Boletales | Basidiomycota | Mlinaric et al., 2005 |
| *Cordyceps militaris ^a,c^* | Hypocreales | Ascomycota | Jiang et al., 2011 |
| *Cortinarius caperatus ^c^* | Agaricales | Basidiomycota | Amoros et al., 1998; Piraino & Brandt 1999 |
| *Cortinarius rubellus ^c^* | Agaricales | Basidiomycota | Amoros et al., 1997 |
|  |  |  |  |
| *Cortinarius sanguineus ^c^* | Agaricales | Basidiomycota | Amoros et al., 1997 |
| *Cryptosporiopsis malicoticis ^c^* | Helotiales | Ascomycota | Krohn et al., 1997 |
| *Cylindrocarpon ianthothele ^b,c^* | Hypocreales | Ascomycota | Singh et al., 2003a |
| *Cystoderma amianthinum^c^* | Agaricales | Basidiomycota | Kandefer-Szerszeń et al., 1980 |
| *Cytospora* sp. | Diaporthales | Ascomycota | Jayasuriya et al., 2003 |
| *Daedalea quercina ^c^* | Polyporales | Basidiomycota | Mlinaric et al., 2005 |
| *Daedaleopsis confragosa* | Polyporales | Basidiomycota | Teplyakova et al., 2012 |
| *Datronia mollis* | Polyporales | Basidiomycota | Teplyakova et al., 2012 |
| *Emericella* sp. | Eurotiales | Ascomycota | Zhang et al., 2011 |
| *Epicoccum nigrum ^c^* | Pleosporales | Ascomycota | Guo et al., 2009 |
| *Exophiala pisciphila ^b^* | Chaetothyriales | Ascomycota | Ondeyka et al., 2003 |
| *Flammulina velutipes ^a,c^* | Agaricales | Basidiomycota | Wang & Ng 2001; Krupodorova et al., 2014 |
| *Fomes fomentarius ^a, c^* | Polyporales | Basidiomycota | Krupodorova et al., 2014 |
| *Fomitopsis pinicola^c^* | Polyporales | Basidiomycota | Mlinaric et al., 2005 |
| *Fusarium heterosporum ^a,c^* | Hypocreales | Ascomycota | Hazuda et al., 1999 |
| *Fusarium* sp. *^c^* | Hypocreales | Ascomycota | Singh et al., 2003b |
| *Galiella rufa ^c^* | Pezizales | Ascomycota | Pérez et al., 2014 |
| *Ganoderma colossum ^c^* | Polyporales | Basidiomycota | El Dine et al., 2008 |
| *Ganoderma lucidum ^a,c^* | Polyporales | Basidiomycota | El Mekkawy et al., 1998; Min et al., 1998; Eo et al., 1999a,b, 2000; Kim et al., 2000; Iwatsuki et al., 2003; Krupodorova et al., 2014; Razumov et al., 2010; Zhang et al., 2014 |
| *Ganoderma pfeifferi ^c^* | Polyporales | Basidiomycota | Mothana et al., 2003; Niedermeyer et al., 2005 |
| *Ganoderma sinense ^c^* | Polyporales | Basidiomycota | Sato et al., 2009 |
| *Gilmaniella humicola ^c^* | uncertain | Ascomycota | Mlinaric et al., 2005 |
| *Gloephyllum trabeum ^c^* | Gloephyllales | Basidiomycota | Mlinaric et al., 2005 |
| *Grifola frondosa ^c^* | Polyporales | Basidiomycota | Gu et al., 2007 |
| *Gymnopilus penetrans ^c^* | Agaricales | Basidiomycota | Kandefer-Szerszeń et al., 1980 |
| *Hymenula cerealis ^c^* | uncertain | Ascomycota | Mlinaric et al., 2005 |
| *Hypholoma fasciculare ^c^* | Agaricales | Basidiomycota | Amoros et al., 1997 |
| *Hypholoma lateritium ^c^* | Agaricales | Basidiomycota | Amoros et al., 1997 |
| *Inonotus hispidus* | Polyporales | Basidiomycota | Awadh Ali et al., 2003 |
| *Inonotus obliquus ^c^* | Polyporales | Basidiomycota | Ichimura et al., 1998 |
| *Inonotus tamaricis ^c^* | Polyporales | Basidiomycota | Singh et al., 2003a |
| *Ishnoderma benzoinum* | Polyporales | Basidiomycota | Teplyakova et al., 2012 |
| *Lactarius mitissimus ^c^* | Russulales | Basidiomycota | Kandefer-Szerszeń et al., 1980 |
| *Lactarius necator ^c^* | Russulales | Basidiomycota | Kandefer-Szerszeń et al., 1980 |
| *Lactarius torminosus ^c^* | Russulales | Basidiomycota | Amoros et al., 1997 |
| *Lactarius volemus* | Russulales | Basidiomycota | Kandefer-Szerszeń et al., 1980 |
| *Laetiporus sulphureus ^c^* | Polyporales | Basidiomycota | Mlinaric et al., 2005 |
| *Laricifomes officinalis* | Polyporales | Basidiomycota | Teplyakova et al., 2012 |
| *Leccinum aurantiacum ^c^* | Boletales | Basidiomycota | Kandefer-Szerszeń et al., 1980 |
| *Lentinula edodes* | Polyporales | Basidiomycota | Tochikura et al., 1988; Suzuki et al., 1989; Sorimachi et al., 1990; Ngai & Ng 2003; Razumov et al., 2010; Rincão et al., 2012, Kruporodova et al., 2014; Matsuhisa et al., 2015 |
| *Leptographium lundbergii^a,c^* | Ophiostomatales | Ascomycota | Mlinaric et al., 2005 |
| *Lenzites betulina* | Polyporales | Basidiomycota | Teplyakova et al., 2012 |
| *Lyophyllum shimeji ^c^* | Agaricales | Basidiomycota | Krupodorova et al., 2014 |
| *Macrocystidia cucumis ^c^* | Agaricales | Basidiomycota | Saboulard et al., 1998 |
| *Macrolepiota procera ^c^* | Agaricales | Basidiomycota | Kandefer-Szerszeń et al., 1980 |
| *Marasmius oreades ^c^* | Agaricales | Basidiomycota | Kandefer-Szerszeń et al., 1980 |
| *Mycena pura ^a,^ ^c^* | Agaricales | Basidiomycota | Kandefer-Szerszeń et al., 1980 |
| *Mycena zephirus ^c^* | Agaricales | Basidiomycota | Kandefer-Szerszeń et al., 1980 |
| *Hypholoma fasciculare ^c^* | Agaricales | Basidiomycota | Kandefer-Szerszeń et al., 1980 |
| *Neosartorya udagawae* | Eurotiales | Ascomycota | Yu et al., 2016 |
| *Neosartorya* sp. | Eurotiales | Ascomycota | Singh et al., 2003a |
| *Nigrospora* sp. | Xylariales | Ascomycota | Zhang et al., 2016 |
| *Omphalatus illudens ^c^* | Agaricales | Basidiomycota | Lehmann et al., 2003 |
| *Paxillus involutus ^a,^ ^c^* | Boletales | Basidiomycota | Kandefer-Szerszeń et al., 1980 |
| *Penicillium brevicompactum ^a^* | Eurotiales | Ascomycota | Sebastian et al., 2011 |
| *Penicillium chrysogenum ^a^* | Eurotiales | Ascomycota | Peng et al., 2014 |
| *Penicillium multicolor ^a^* | Eurotiales | Ascomycota | Matsuzaki et al., 1995 |
| *Penicillium* sp. | Eurotiales | Ascomycota | Omura et al., 1993; Singh et al., 2003a; Shiomi et al., 2005; Raekiansyah et al., 2017 |
| *Periconia* sp. | Pleosporales | Ascomycota | Zhang et al., 2015 |
| *Pestalotiopsis theae ^a^* | Amphisphaeriales | Ascomycota | Li et al., 2008 |
| *Pestalotiopsis vaccinia ^a,c^* | Amphisphaeriales | Ascomycota | Wang et al., 2014a |
| *Pestalotiopsis* sp. | Amphisphaeriales | Ascomycota | Jia et al., 2015 |
| *Pezicula livida ^a,c^* | Helotiales | Ascomycota | Krohn et al., 1997 |
| *Phialophora mutabilis ^a,c^* | Chaetothyriales | Ascomycota | Mlinaric et al., 2005 |
| *Pholiota adiposa ^a,^ ^c^* | Agaricales | Basidiomycota | Mlinaric et al., 2005 |
| *Phoma* sp.*^c^* | Pleosporales | Ascomycota | Hazuda et al., 1999 |
| *Phomopsis* sp. | Diaporthales | Ascomycta | Bunyapaiboonsri et al., 2010 |
| *Pichia anomala ^a,c^* | Saccharomycetales | Ascomycota | Mlinaric et al., 2005 |
| *Plectophomella* sp. | incertae sedis | Ascomycota | Bunyapaiboonsri et al., 2010 |
| *Pleurotus cornucopiae var. citrinopileatus ^a^* | Agaricales | Basidiomycota | Razumov et al., 2010 |
| *Pleurotus eryngii ^c^* | Agaricales | Basidiomycota | Kruporodova et al., 2014 |
| *Pleurotus ostreatus ^a, c^* | Agaricales | Basidiomycota | Wang & Ng 2000; Razumov et al., 2010; Gao et al., 2013, Kruporodova et al., 2014 |
| *Rhodonia placenta ^c^* | Polyporales | Basidiomycota | Mlinaric et al., 2005 |
|  |  |  |  |
| *Pullularia* sp.*^c^* | Dothideales | Ascomycota | Isaka et al., 2007 |
| *Ramaria apiculate ^a, c^* | Gomphales | Basidiomycota | Kandefer-Szerszeń et al., 1980 |
|  |  |  |  |
| *Russula fellea* | Russulales | Basidiomycota | Kandefer-Szerszeń et al., 1980 |
| *Russula paludosa* | Russulales | Basidiomycota | Wang et al., 2007 |
| *Schizophyllum commune ^c^* | Agaricales | Basidiomycota | Mlinaric et al., 2005; Kruporodova et al., 2014 |
| *Scleroderma citrinum* | Boletales | Basidiomycota | Kanokmedhakul et al., 2003 |
| *Scytalidium* sp.*^a,c^* | Helotiales | Ascomycota | Rowley et al., 2003 |
| *Serpula lacrymans ^c^* | Boletales | Basidiomycota | Mlinaric et al., 2005 |
| *Sistotrema brinkmannii ^c^* | Cantharellales | Basidiomycota | Mlinaric et al., 2005 |
| *Stachybotrys chartarum ^a^* | Hypocreales | Ascomycota | Li et al., 2014; Ma et al., 2013; Zhao et al., 2017 |
| *Stachybotrys nephospora ^a^* | Hypocreales | Ascomycota | Sawadjoon et al., 2004 |
| *Stachybotrys* sp. | Hypocreales | Ascomycota | Yoshimoto et al., 1999; Minagawa et al., 2002 |
| *Stereum hirsutum ^c^* | Russulales | Basidiomycota | Mlinaric et al., 2005 |
| *Stereum rugosum ^c^* | Russulales | Basidiomycota | Mlinaric et al., 2005 |
| *Stereum submentosum ^c^* | Russulales | Basidiomycota | Mlinaric et al., 2005 |
| *Talaromyces flavus ^a,b^* | Eurotiales | Ascomycota | Singh et al., 2003a |
| *Trametes gibbosa ^c^* | Polyporales | Basidiomycota | Amoros et al. 1997, Mlinaric et al., 2005; Teplyakova et al., 2012 |
| *Trametes versicolor ^c^* | Polyporales | Basidiomycota | Okada & Minamishima 1987; Tochikura et al., 1987; Collins & Ng 1997; Teplyakova et al., 2012; Krupodorova et al., 2014 |
| *Trichoderma harzianum ^a,c^* | Hypocreales | Ascomycota | Mlinaric et al., 2005 |
| *Trichoderma* sp. | Hypocreales | Ascomycota | Pang et al., 2018 |
| *Tricholoma acerbum* | Agaricales | Basidiomycota | Amoros et al., 1997 |
| *Tricholoma virgatum* | Agaricales | Basidiomycota | Amoros et al., 1997 |
| *Tricholoma portentosum* | Agaricales | Basidiomycota | Kandefer-Szerszeń et al., 1980; Amoros et al., 1997 |
| *Tricholomopsis rutilans* | Agaricales | Basidiomycota | Kandefer-Szerszeń et al., 1980 |
| *Verticillium lecanii ^a,c^* | Glomerellales | Ascomycota | Mlinaric et al., 2005 |
| *Xylaria mellisii ^a,c^* | Xylariales | Ascomycota | Pittayakhajonwut et al., 2005 |
| *Xylaria* sp.*^a,c^* | Xylariales | Ascomycota | Hazuda et al., 1999 |

* Species names used in the table have been amended to the current accepted name for the taxon declared in the cited studies. In cases where the declared taxon has been revised in such a way as to make the conspecificity of the material ambiguous, or the current taxonomy is known to be markedly confused and lack an accurate taxonomic basis, the name is followed by ^a^. When the material used has been identified based on only macro- and/or micromorphological features, but without molecular markers, or vice versa, the name is followed by ^b^. If there are no identification methods reported in cited original studies, the name is followed by ^c^.
