# Supplementary material for: Antiviral Agents From Fungi: Diversity, Mechanisms and Potential Applications
Source: Front Microbiol. 2018 Oct 2;9:2325. doi: 10.3389/fmicb.2018.02325 (PMC6176074; doi:10.3389/fmicb.2018.02325)
Supplement: Supplementary file 2 [file Table_2.DOCX]

**Supplementary table 2.** Reported methods of species identification and evaluation of reproducibility of previous antiviral studies on genus *Ganoderma*.

| **Reference** | **Claimed species, verbatim** | **Material acquired** | **Method of identification** | | **Material specimens deposited** | **Voucher number** | | **Strain number for cultivated material** | **Reproducibility of the experimental set-up** | |  |
| --- | --- | --- | --- | --- | --- | --- | --- | --- | --- | --- | --- |
| El Dine et al., 2008 | *Ganoderma colossum* | Cultivated basidiomes. | Methods not reported. | Toyama University, Japan. | | TMPW 25804 | No number given, but provenance described. | | | Good. | |
| El Mekkawy et al., 1998 | *G. lucidum* (Leyss. ex Fr.) Karst | Basidiomes from Alps Chemical Industries Co. Japan, and Lingzhi General Institute Co. Ltd, Tokyo. | Methods not reported. | Toyama University, Japan. | | Voucher number not given. | Strain not reported. | | | Requires the examination of herbarium specimens. | |
| Eo et al., 1999a | *G. lucidum* (Fr.) Karst | Cultivated basidiomes, bought from herbal drug store. | Methods not reported. | Seoul National Uni. | | No. Cpm 605 | Strain not reported. | | | Requires examination of herbarium specimens. | |
| Eo et al., 1999b | *G. lucidum* (Fr.) Karst. | Cultivated basidiomes, bought from herbal drug store. | Methods not reported. | Seoul National Uni. | | No. Cpm 605 | Strain not reported. | | | Requires the examination of herbarium specimens. | |
| Eo et al., 2000 | *G. lucidum* (Fr.) Karst. | Cultivated basidiomes, bought from herbal drug store. | Methods not reported. | Seoul National Uni. | | No. Cpm 605 | Strain not reported. | | | Requires the examination of herbarium specimens. | |
| Iwatsuki et al., 2003 | *G. lucidum* KARST | Cultivated basidiomes, bought from Kinokuniya Kan-Yaku Kyoku Co. (Tokyo), cultivated in Gunma, Japan | By a description in a medicinal mushroom encyclopedia. | College of Science and Technology, Nihon Uni. | | Voucher number not given. | Strain not reported. | | | Requires the examination of herbarium specimens. | |
| Kim et al., 2000 | *G. lucidum* (Fr.) Karst | Cultivated basidiomes, bought from local store. | Methods not reported. | Seoul National Uni. | | No. Cpm 605 | Strain not reported. | | | Requires the examination of herbarium specimens. | |
| Niedermeyer et al., 2005 | *G. pfeifferi* Bres. | Basidiomes collected from wild, Germany. | Methods not reported. | Ernst-Moritz-Arndt Universität, Greifswald. | | Voucher number given. | PI-28 | | | Good. | |
| Min et al., 1998 | *G. lucidum* Karst. | Cultivated basidiomes, from China, Vietnam and Japan. Spores, without provenance. Above from Lingzhi General Institute Co. Ltd. Tokyo. Antler shaped basidiomes from Seizen Co. Kyoto. | Methods not reported. | No report of specimens deposited. | | Voucher number not given. | Strain not reported. | | | Requires identical samples. | |
| Mothana et al., 2003 | *G. pfeifferi* Bres. | Basidiomes collected from wild, Germany. | Methods not reported. | Ernst-Moritz-Arndt Universität, Greifswald. | | PI-28 | - | | | Good. | |
| Sato et al., 2009 | *G. sinense* | Bought from market, Chengdu, China. | Method not reported. | Toyama University, Japan. | | Voucher number not given. | Strain not reported. | | | Requires the examination of herbarium specimens. | |
| Zhang et al., 2014 | *G. lucidum* | Crude “Reishi” extract, from Kunming Institute of Botany, Chinese Academy of Science. | Methods not reported. | No report. | | Voucher number not given. | Strain not reported. | | | Requires identical samples. | |
| Liu et al., 2004 | *G. lucidum* (Fr.) Karst / Karst | Live strain from the authors lab. | Methods not reported. | No report. | | Voucher number not given. | Strain not reported. | | | Good, if the strain used is alive. Low, if not. | |
